# A targeted proteomics screen reveals serum and synovial fluid proteomic signature in patients with gout

**DOI:** 10.3389/fimmu.2024.1468810

**Published:** 2024-11-14

**Authors:** Zhengping Huang, Xiaoyan Zhong, Yuexi Zhang, Xinjian Li, Meng Liu, Yukai Huang, Jian Yue, Guanqun Yi, Hongji Liu, Bingyan Yuan, Xu Chen, Shaoling Zheng, Tianwang Li

**Affiliations:** ^1^ The Second School of Clinical Medicine, Southern Medical University, Guangzhou, China; ^2^ Department of Rheumatology and Immunology, The Affiliated Guangdong Second Provincial General Hospital of Jinan University, Guangzhou, China; ^3^ Department of Rheumatology and Immunology, Zhaoqing Central People’s Hospital, Zhaoqing, China

**Keywords:** crystal arthropathies, osteoarthritis, biomarkers, synovial fluid analysis, proteomics, cytokines and inflammatory mediators

## Abstract

**Objective:**

To characterize the inflammatory proteome in both serum and synovial fluid (SF) of patients with gout, in comparison to healthy controls and individuals with osteoarthritis (OA), by utilizing a high-quality, high-throughput proteomic analysis technique.

**Methods:**

Using the Olink Target 48 Inflammation panel, we measured serum concentrations of 45 inflammatory proteins in gout, OA, and healthy controls. We analyzed protein levels in SF samples from gout and OA, performed ROC curve analyses to identify diagnostic biomarkers, evaluate efficacy, and set cut-off values. Additionally, A protein-protein interaction (PPI) network was used to study protein relationships and significance.

**Results:**

We have delineated the proteomic landscape of gout and identified 20 highly differentially expressed proteins (DEPs) in the serum of gout patients in comparison to that of healthy controls, which included VEGF-A, MMP-1, TGF-α, and OSM with corresponding area under the curve (AUC) values of 0.95, 0.95, 0.92, and 0.91 respectively. For the analysis of synovial fluid, 6 proteins were found to be elevated in gout in contrast to osteoarthritis (OA), among which IP-10, VEGF-A, IL-8, and MIP-3β had corresponding AUC values of 0.78, 0.78, 0.76, and 0.75 respectively. The protein-protein interaction (PPI) network analysis identified significantly prominent pathways in gout.

**Conclusion:**

This research marks a significant advancement in elucidating the inflammatory profile present in the serum and synovial fluid of individuals suffering from gout. Our discoveries have identified several novel proteins in both serum and synovial fluid that are potential biomarkers for diagnostic purposes and are believed to have critical roles as pathogenic factors in the pathophysiology of gout.

## Introduction

Gout is among the most prevalent forms of arthritis, defined by an innate immune response that is activated due to hyperuricemia. This condition is aggravated by the build-up of monosodium urate crystals (MSU) within joints and surrounding connective tissues (1). Epidemiological research conducted recently has highlighted a swift and widespread rise in the incidence of both primary gout and hyperuricemia on a global scale (2). Specifically, in China, the adjusted prevalence rate for gout stands at 3.2%, with hyperuricemia impacting a significant 17.7% of the population (3).

The accumulation of monosodium urate (MSU) crystals in the joint triggers an innate immune reaction, where these crystals are recognized as foreign bodies by the immune system (4). This recognition leads to the activation of neutrophils, a type of white blood cell. Central to the cellular inflammatory cascade initiated by MSU crystals is the NLRP3 inflammasome (5). Once activated, the NLRP3 inflammasome promotes the secretion of pro-inflammatory cytokines, including interleukin-1 beta (IL-1β) and interleukin-18 (IL-18). Among these, IL-1β is a crucial factor in mediating the inflammatory cascade characteristic of gout (6). The secretion of these cytokines initiates the recruitment of additional immune cells, setting off an inflammatory cascade. This cascade manifests as the hallmark symptoms of gout, such as intense pain, redness, swelling, and heat in the affected joint. While the activation of the NLRP3 inflammasome is recognized as a critical event, the exact mechanisms driving gout remain incompletely understood. It is thought that a complex interplay of multiple regulatory pathways contributes to the diverse clinical manifestations observed in gouty inflammation.

The Proximity Extension Assay (PEA) represents a cutting-edge proteomics approach, meticulously quantifying even the most minute protein concentrations within biological fluids, including as little as 1μl of plasma or serum (7). Utilizing the PEA technology, Olink reagents employ a set of 45 antibody probe pairs, each labeled with oligonucleotides and specifically designed to bind to their corresponding target proteins within the sample (8). This method is further enhanced by PCR-based signal amplification, which markedly boosts sensitivity and facilitates the detection of proteins at ultra-low levels.

To our knowledge, this study is the first to delineate the inflammatory profile of both serum and synovial fluid (SF) in gout patients by examining a comprehensive panel of 45 inflammatory proteins via PEA technology. Our objective was to map the inflammatory proteome of gout patient serum and SF, with the goal of identifying novel proteins that could serve as potential biomarkers for diagnosis, as well as to identify proteins that may play significant roles in the underlying mechanisms of gout pathogenesis.

## Methods

### Human subjects

Research was granted ethical approval by the Ethics Committee of Guangdong Second Provincial General Hospital under the protocol number 2024-KY-KZ-285-01. Informed consent was obtained from all participants prior to the collection of research samples. Clinical data were gathered through a retrospective review of medical records. Gout patients were selected based on the 2015 American College of Rheumatology (ACR)/European League Against Rheumatism (EULAR) classification criteria for gout, while osteoarthritis (OA) patients were classified according to the clinical criteria established by the ACR (9, 10). All patients, both with gout and OA, exhibited active disease as indicated by elevated C-reactive protein (CRP) levels, clinical symptoms, and findings from a physical examination conducted by a rheumatologist. Serum and SF samples were collected from patients with active gout and osteoarthritis (OA) during their inpatient or outpatient visits to the Department of Rheumatology and Clinical Immunology. Healthy control serum samples were obtained from volunteer participants. All collected samples were stored at -80°C and were thawed immediately prior to assay preparation.

### PEA

We utilized the Olink Target 48 Cytokine Panel to analyze 45 analytes in plasma and serum samples, adhering to the manufacturer’s protocol. Each assay run involved processing forty samples on a 48 × 48 integrated fluidic circuit (IFC). The samples were incubated with 1 μl of plasma or serum and antibodies for 18 hours. Following hybridization, DNA tags were extended and preamplified using a Bio-Rad T100 Thermocycler (Hercules, CA). The preamplified samples, along with primers, were then loaded onto a primed IFC, and quantitative PCR (qPCR) amplification was performed for 40 cycles on an Olink Signature Q-100 instrument. To maintain accuracy, each run included internal controls and calibrators. Data analysis and quality control were performed using the Olink NPX Signature software, which also facilitated the conversion of NPX-values to protein concentrations in standard units (pg/mL) by fitting them to a standard curve.

### Bioinformatics and statistical analysis

For the generation of PCA, heatmap, dotplot, ggplot, and volcano plots, the seurat package (version 4.0.1), complexheatmap package (version 2.6.2), ggrepel package (version 0.9.2), and ggplot2 package (version 3.3.3) were utilized within the R programming environment (version 4.1.2). Additionally, STRING (Search Tool for the Retrieval of Interacting Genes/Proteins) functional protein association networks and signal analysis were created using the STRING online tool (http://string-db.org/, accessed on 10 July 2024). Independent samples were analyzed using T-tests or the Mann–Whitney U tests. For contingency analysis involving more than two groups, one-way independent analysis of variance or Kruskal–Wallis tests were conducted for continuous variables. A P-value of less than 0.05 was deemed statistically significant, unless indicated otherwise. For differentially expressed proteins (DEPs) between two groups, A p-value of less than 0.05 and an absolute log2 fold change greater than 1 were defined as criteria for significant differential expression. Receiver Operating Characteristic (ROC) curve analysis was employed to assess the sensitivity and specificity of potential biomarkers across a range of threshold values. All statistical analyses, including ROC curve evaluations, were carried out using Prism 8.0 software (GraphPad Software, La Jolla, CA).

## Results

### Overall proteomic signature

**Figure 1:**
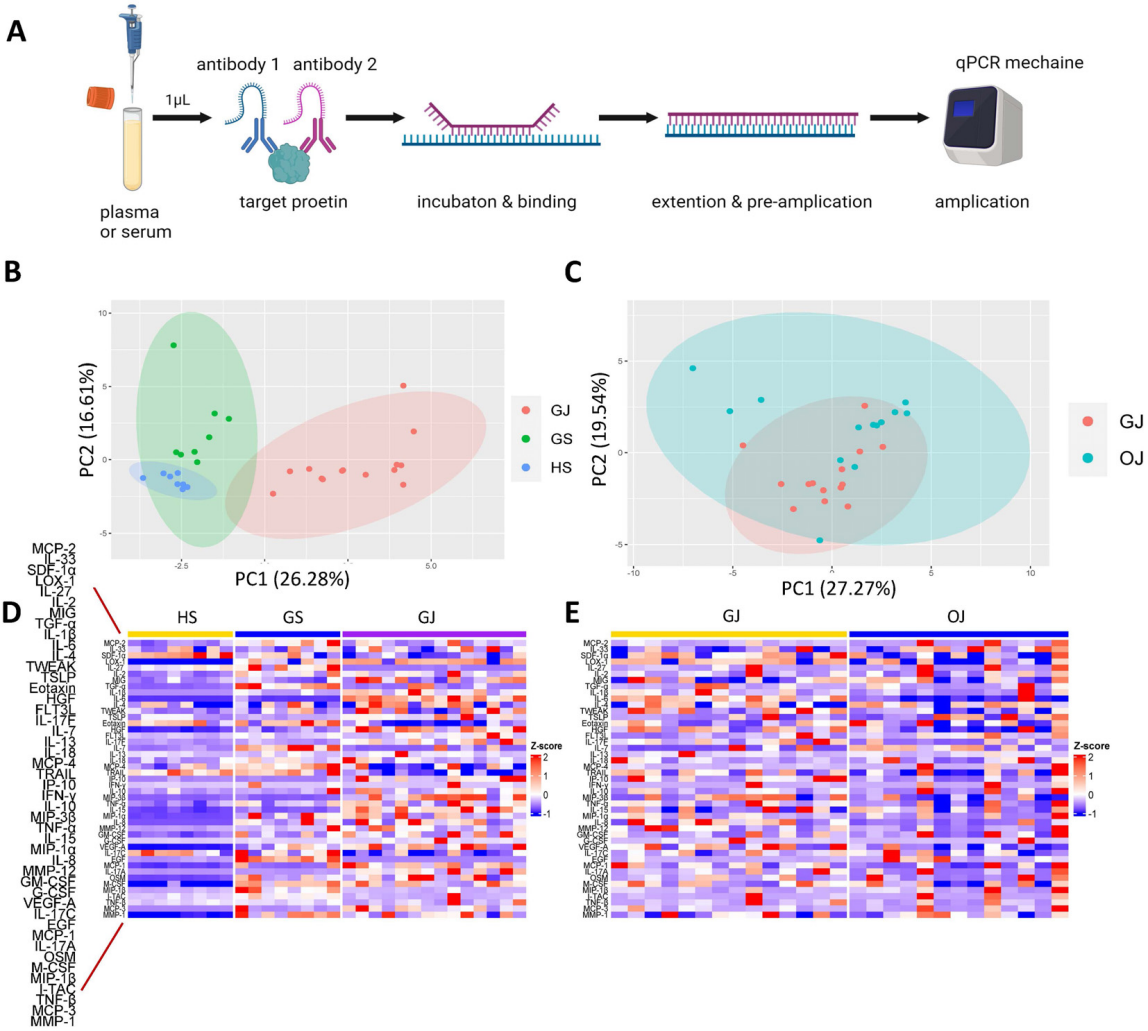
Comprehensive proteomic profile of gout. **(A)** Schematic representation of the key steps in PEA. **(B)** PCA of serum proteins in groups GS, GJ, and HS. Each data point corresponds to a sample, with PC1 represented on the vertical axis and PC2 on the horizontal axis. **(C)** PCA of serum proteins in groups GJ and OJ. Each data point represents a sample, with PC1 shown on the vertical axis and PC2 on the horizontal axis. **(D)** Heatmap depicting serum protein expression levels in groups GS, GJ, and HS, where blue indicates lower expression and red indicates higher expression. **(E)** Heatmap illustrating serum protein expression levels in groups GJ and OJ, with blue indicating lower expression and red indicating higher expression.

In total, serum samples were collected from 8 gout patients (GS), 8 OA patients (OS), and 8 healthy controls (HS). Additionally, synovial fluid (SF) was collected from 14 gout patients (GJ) and 13 OA patients (OJ) (**Supplementary Table S1**). Subsequently, the Olink Target 48 Cytokine Panel, which measures 45 proteins such as cytokines, chemokines, and growth factors, was used (**Table 1**). The key steps of PEA are illustrated in **Figure 1A**. To depict the overall population structure and variance of the samples, Principal Component Analysis (PCA) was utilized. PCA of serum proteins revealed distinct clustering patterns among gout, OA, and healthy controls (HC), effectively segregating them into their respective groups (**Figure 1B**). The PCA plot demonstrated a high level of consistency within the protein profiles of the healthy controls, while the protein profiles of gout serum samples exhibited significant heterogeneity. PCA analysis of SF proteins indicated substantial overlap between gout and OA, suggesting a similarity in proteomic signatures within these groups (**Figure 1C**). A heatmap was then used to show the expression levels of various proteins among the groups. In GS, several proteins displayed different expression levels compared to HS, with some proteins exhibiting significantly higher levels (**Figure 1D**). Similarly, there were notable differences in protein levels between the GJ and HS groups. In the SF analysis, GJ did not show many significantly higher levels of proteins compared to OJ, which aligned with the results from the PCA plot (**Figure 1E**).

**Table 1:** 45 inflammatory proteins in the Olink Target 48 Inflammation panel.

		
VEGF-A	M-CSF	IL-1β
TWEAK	MCP-4	IL-18
TSLP	MCP-3	IL-17F
TRAIL	MCP-2	IL-17C
TNF-β	MCP-1	IL-17A)
TNF-α	LOX-1	IL-15
TGF-α	I-TAC	IL-13
SDF-1α	IP-10	IL-10
OSM	IL-8	IFN-γ
MMP-12	IL-7	HGF
MMP-1	IL-6	GM-CSF
MIP-3β	IL-4	G-CSF
MIP-1β	IL-33	FLT3L
MIP-1α	IL-27	Eotaxin
MIG	IL-2	EGF

### Profiling of gout versus controls samples

**Figure 2:**
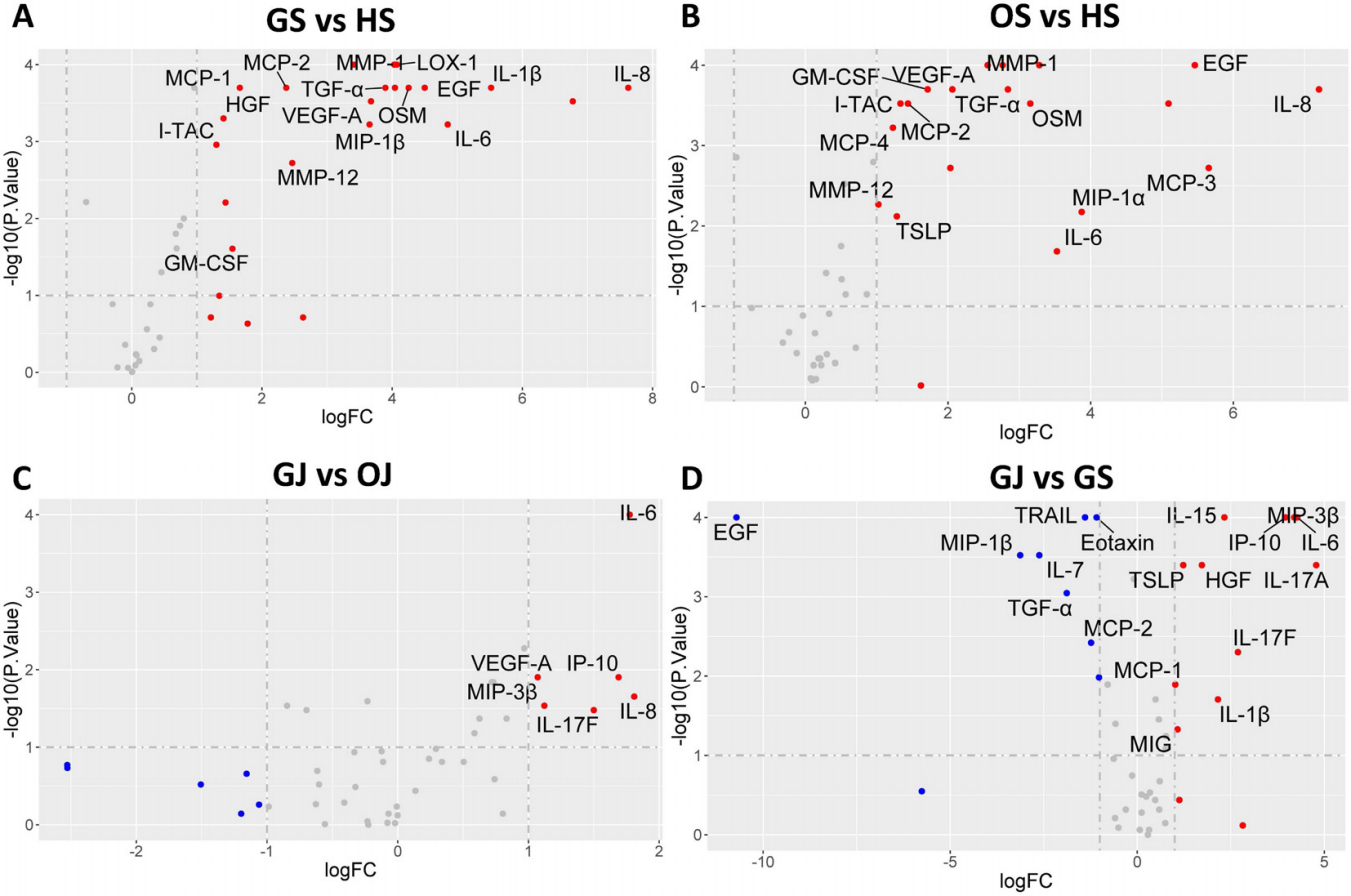
Volcano plot illustrating differential protein levels between two subject groups. **(A)** DEPs in serum between gout patients and healthy controls. **(B)** DEPs in serum between OA patients and healthy controls. **(C)** DEPs in SF between gout patients and healthy controls. **(D)** DEPs between serum and SF in gout patients. Each data point corresponds to a protein, with the horizontal axis representing the protein’s fold change magnitude on a log2 scale. The vertical axis indicates significance, presented as the -log10 transformation of the P value. Proteins located on the right side of the plot indicate higher levels in the respective group’s samples.

In order to elucidate the expression patterns of inflammatory proteins among individuals with gout and to compare these with cytokine levels in different groups, we conducted an analysis of protein levels across the groups. A comparative analysis between GS and HS revealed that 20 proteins are markedly increased in GS, including IL-8, MCP-3, IL-1β, IL-6, EGF, OSM, LOX-1, MIP-1α, MMP-1, TGF-α, VEGF-A, MIP-1β, IL-7, MMP-12, MCP-2, MCP-1, GM-CSF, IL-2, HGF and I-TAC (**Figure 2A**). Most of these proteins were also observed to be significantly higher in OS compared to HS, except for IL-2, HGF, and MCP-1 (**Figure 2B**). In the comparison between GJ and OJ, the levels of IL-8, IL-6, IP-10, IL-17F, MIP-3β, and VEGF-A were found to be elevated in GJ (**Figure 2C**). When comparing GJ to GS, a distinct pattern of protein expression was observed. A set of proteins, including IL-17A, IL-6, MIP-3β, IP-10, IL-17F, IL-15, IL-1β, HGF, TSLP, MIG, and MCP-1, were found to be significantly higher in GJ. In contrast, the levels of IL-17C, Eotaxin, MCP-2, TRAIL, TGF-α, IL-7, MIP-1β, IL-27, and EGF were significantly higher in GS (**Figure 2D**).

**Figure 3:**
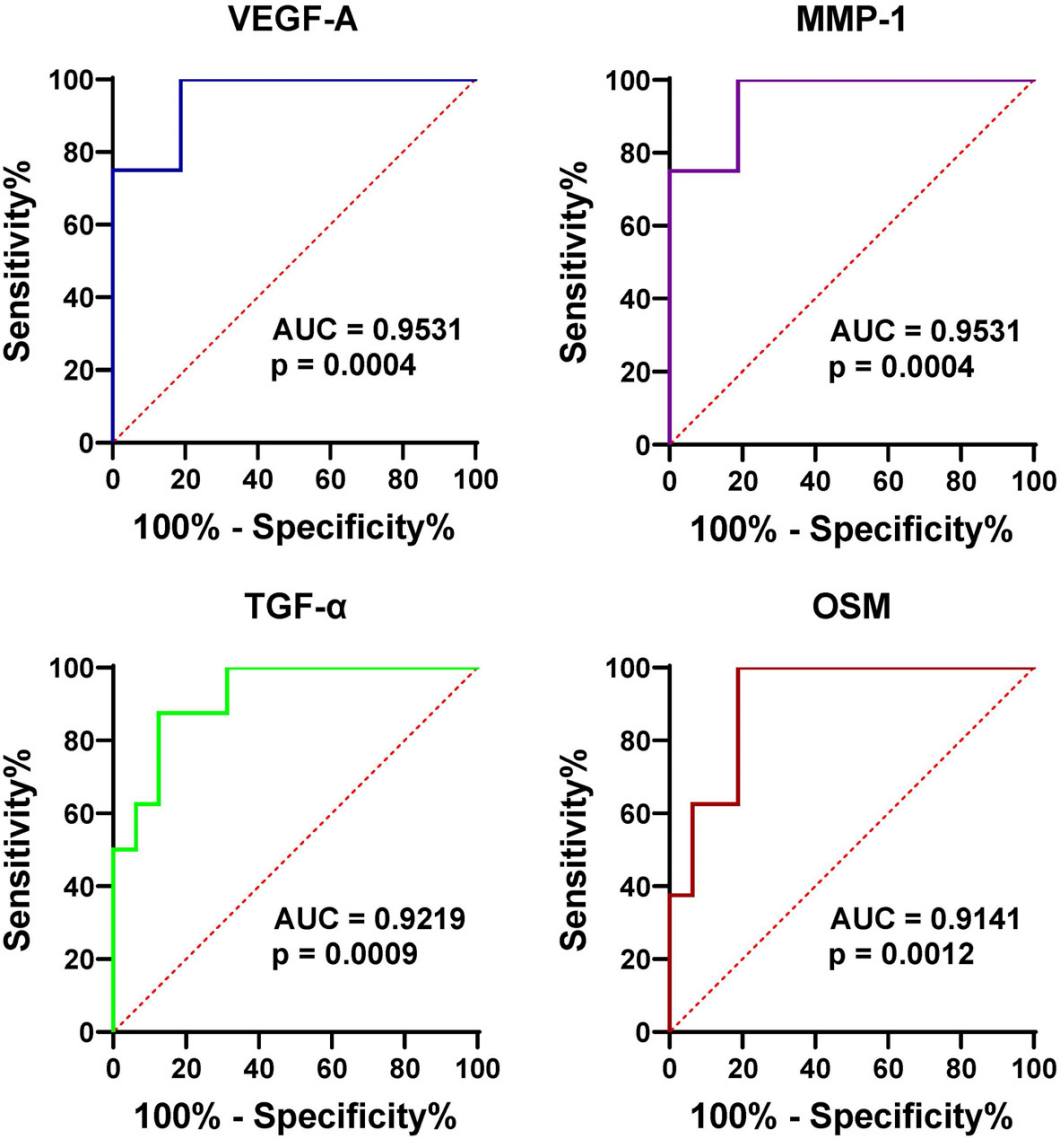
ROC curve analysis assessing the diagnostic potential of serum VEGF-1, MMP-1, TGF-α, and OSM in differentiating gout from OA and healthy controls. This figure presents ROC curves for the four proteins (VEGF-1, MMP-1, TGF-α, and OSM) in serum, demonstrating their ability to distinguish gout patients from those with OA and healthy controls. The AUC values are provided for each protein, indicating their diagnostic accuracy.

**Figure 4:**
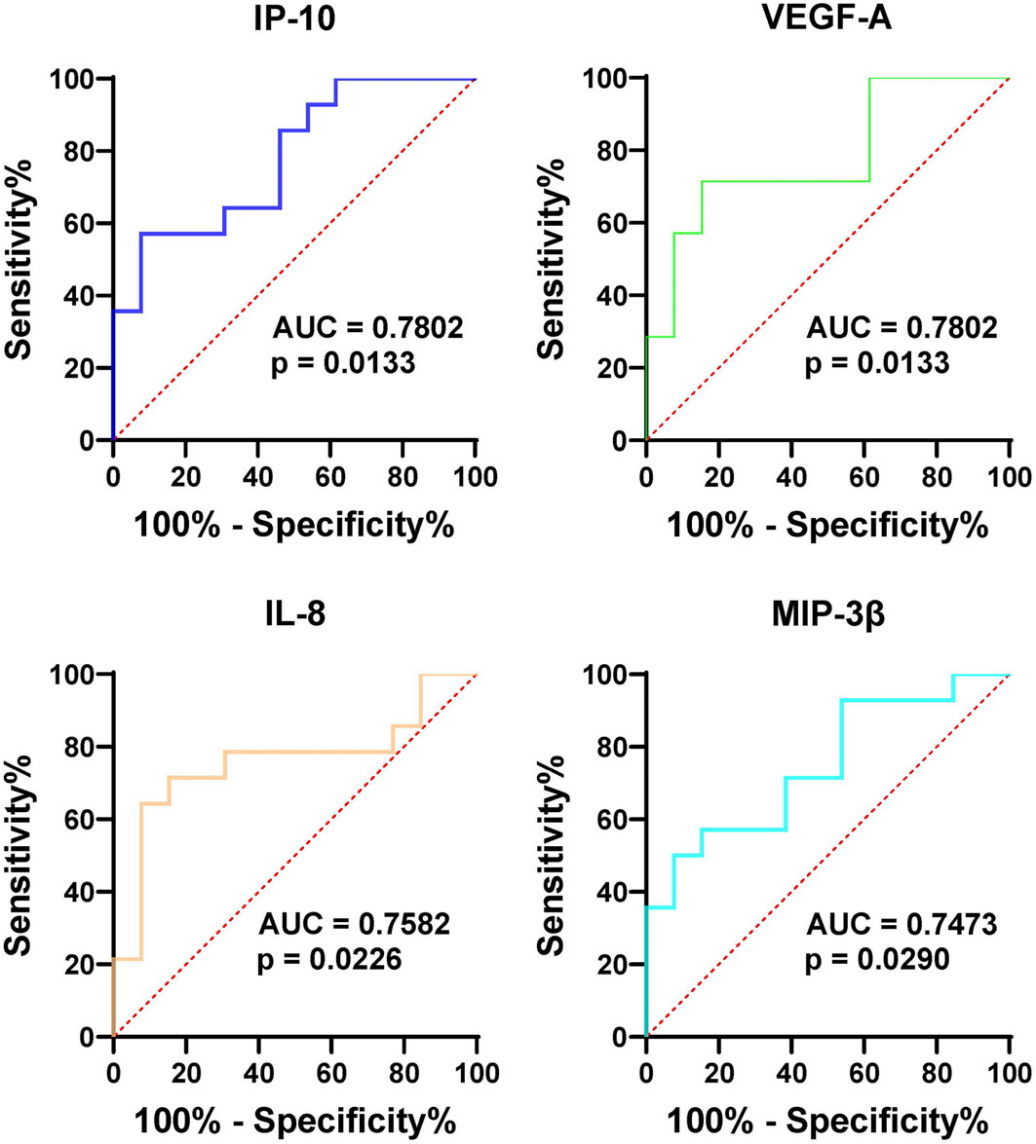
ROC curve analysis assessing the diagnostic potential of SF VEGF-1, MMP-1, TGF-α, and OSM in differentiating gout from OA and healthy controls. Similar to **Figure 3**, this figure shows ROC curves for the four proteins in synovial fluid (SF), indicating their potential as diagnostic markers for distinguishing gout from OA and healthy controls. The AUC values are provided for each protein, reflecting their diagnostic performance.

To further assess the effectiveness of these proteins in distinguishing gout from OA and healthy controls, we performed ROC curve analysis. The proteins that were identified to be highly expressed in GS and GJ than in the control group were selected for inclusion in the ROC analysis. In the serum samples, the proteins which showed higher expression levels in GS compared with OS and HS samples included VEGF-A, MMP-1, TGF-α, and OSM, with the corresponding area under the curve (AUC) values of 0.95, 0.95, 0.92, and 0.91 respectively (**Figure 3**). The determined cut-off values for VEGF-A, MMP-1, TGF-α, and OSM were 674.1 pg/mL, 2169 pg/L, 25.83 pg/L, and 13.23 pg/L. In the synovial fluid (SF) samples, the proteins presenting elevated expression in GJ as opposed to OJ samples were IP-10, VEGF-A, IL-8, and MIP-3β, with the AUC values of 0.78, 0.78, 0.76, and 0.75 respectively (**Figure 4**). The identified cut-off values for IP-10, VEGF-A, IL-8, and MIP-3β were 226.0 pg/mL, 2387.0 pg/L, 148.6 pg/L, and 587.1 pg/L.

### Protein-protein interaction and pathway enrichment analysis

**Figure 5:**
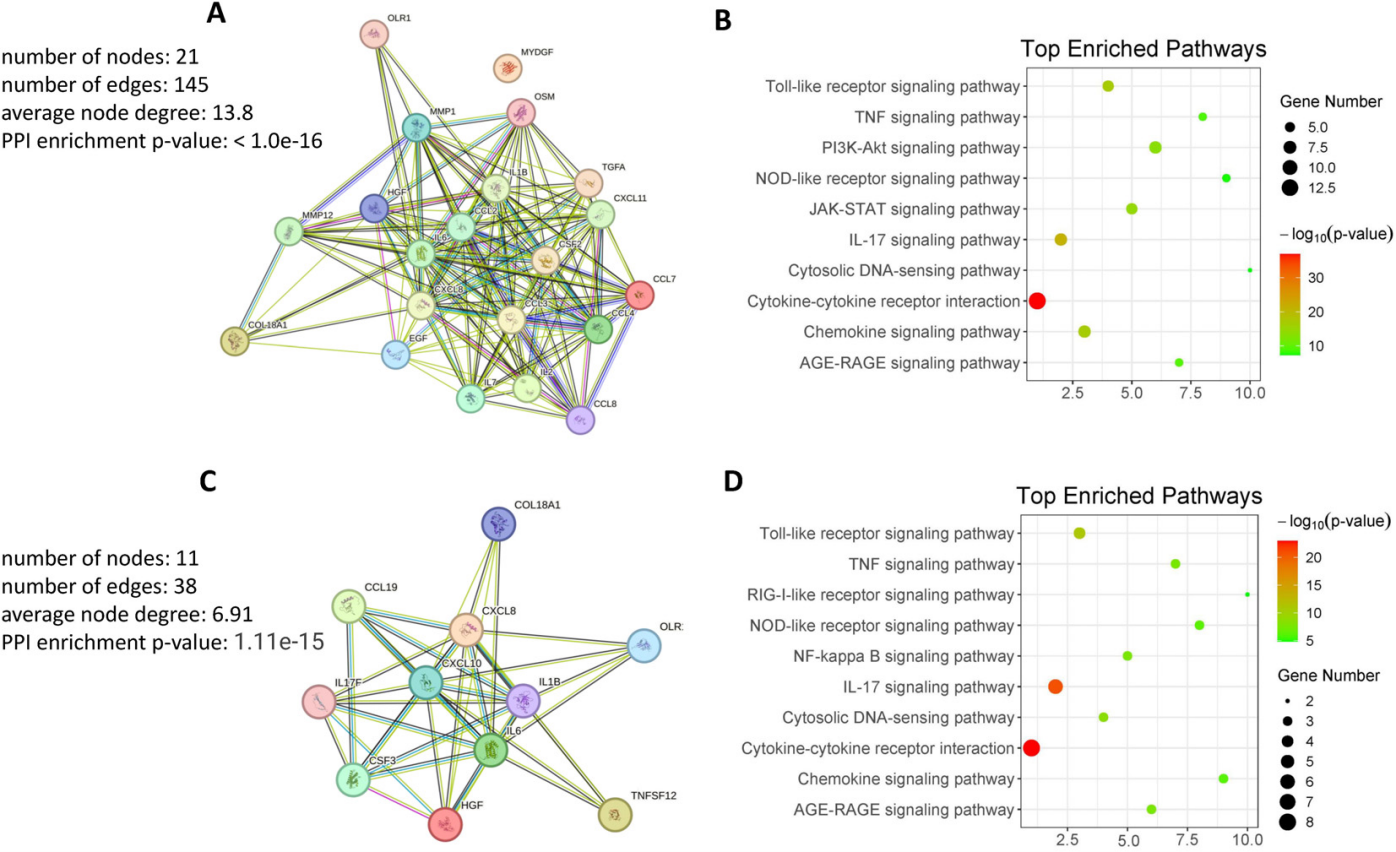
Protein-protein interaction and pathway enrichment analysis. **(A)** Protein-Protein Interaction Network of DEPs in GS compared to HS. The network visualizes interactions among DEPs, highlighting key nodes and pathways. **(B)** KEGG Pathway Enrichment Analysis of Highly DEPs in GS compared to HS. The analysis identifies enriched pathways, providing insights into the biological processes involved. **(C)** Protein-Protein Interaction Network of Highly DEPs in GJ compared to OJ. The network visualizes interactions among DEPs, highlighting key nodes and pathways. **(D)** KEGG Pathway Enrichment Analysis of Highly DEPs in GJ compared to OJ. The analysis identifies enriched pathways, providing insights into the biological processes involved.

To understand the functional relationships and biological significance of proteins in gout. A protein-protein interaction (PPI) network was constructed using the STRING (Search Tool for the Retrieval of Interacting Genes/Proteins) online tool (http://string-db.org/, accessed on 20 June 2024) was used to analyze the protein interaction network. Inputting proteins (**Supplementary Table S2**) that show higher expression in GS compared to HS revealed that most of these proteins participate in forming a densely interconnected PPI network (**Figure 5A**). Notably, only one protein (MYDGF) is not part of this network. The resulting network comprises 21 nodes connected by 145 edges, with an average node degree of 13.8 and an average local clustering coefficient of 0.836. The number of edges in this network exceeds what would be expected by random associations, indicating a significantly higher number of interactions than expected. This network is characterized by a PPI enrichment p-value of < 10e−16, underscoring the substantial enrichment of interactions (**Figure 5A**). These findings suggest that the majority of proteins with differential expression in GS can interact with each other effectively within this internal PPI network. To clarify the function of differentially expressed proteins in the comparison between GS and HS, a KEGG pathways enrichment analysis was performed. The analysis identified 53 significantly enriched pathways, with the most prominent KEGG pathways visualized in bubble plot (**Figure 5B**). The bubble plot effectively visualizes the most significantly enriched pathways, highlighting key processes such as “Cytokine-cytokine receptor interaction”, “Chemokine signaling pathway”, “IL-17 signaling pathway”, “Toll-like receptor signaling pathway”, “JAK-STAT signaling pathway” and “PI3K-AKT signaling pathway”. These significantly enriched pathways offer valuable insights into the underlying biological mechanisms of gout. In particular, the “Cytosolic DNA-sensing pathway” exhibit smaller dot sizes but remain statistically significant, suggesting their potential relevance to gout.

Subsequently, we examined proteins (**Supplementary Table S2**) that exhibit higher expression in GJ compared to OJ. The data revealed that these proteins are primarily involved in forming a densely interconnected PPI network (**Figure 5C**). The resulting network consists of 11 nodes connected by 38 edges, with an average node degree of 6.91 and an average local clustering coefficient of 0.848. The number of edges in this network surpasses what would be anticipated by random associations, indicating a significantly higher number of interactions than expected. This network is distinguished by a PPI enrichment p-value of 1.11e-15, highlighting the substantial enrichment of interactions. These findings suggest that the majority of proteins with differential expression in GJ can effectively interact with each other within this internal PPI network. Additionally, KEGG pathway enrichment analysis was also conducted using proteins that exhibited higher expression in GJ compared to OJ. The bubble plot provides a clear visualization of the most significantly enriched pathways in GJ (**Figure 5D**). Many of these pathways were also identified in GS compared to HS, including “Cytokine-cytokine receptor interaction,” “IL-17 signaling pathway,” and “Toll-like receptor signaling pathway.” Additionally, pathways such as “Cytosolic DNA-sensing pathway” and “AGE-RAGE signaling pathway” were found to be associated with GJ. These enriched pathways offer valuable insights into the underlying biological mechanisms of arthritis in gout.

## Discussion

Gout is characterized by both acute and chronic inflammatory responses. Cytokines and chemokines play crucial roles in the pathogenesis of arthritis by regulating inflammation, immune responses, immune cells migration and tissue repair within the joints (11). These signaling molecules are key mediators of the complex inflammatory processes involved in various forms of arthritis, including gout. Chemokines such as CCL2 (MCP-1), CCL5 (RANTES), and CXCL8 (IL-8) are involved in recruiting monocytes, T cells, and neutrophils to the synovium, contributing to the perpetuation of inflammation and tissue damage (12–14). In osteoarthritis (OA), TGF-β plays a role in maintaining cartilage homeostasis and promoting chondrocyte proliferation and matrix synthesis (15). However, dysregulation of TGF-β signaling can lead to cartilage degradation and OA progression (15, 16).

It is now widely acknowledged that the proteome offers profound insights into the pathophysiology of inflammatory arthritis, serving as a pivotal resource for the discovery of novel biomarkers (17). PEA, pioneered by Olink Proteomics, stands out as an effective method for the simultaneous identification and quantification of a multitude of biomarkers within a single sample. Here, we utilized high-fidelity protein quantification through PEA to investigate proinflammatory mediators in gout and controls. The PCA plot revealed a high degree of consistency in the protein profiles of the healthy controls. In contrast, the protein profiles of the gout serum samples showed significant heterogeneity. Additionally, the PCA analysis of SF proteins indicated a substantial overlap between gout and OA, suggesting a resemblance in proteomic signatures within these groups. Furthermore, Olink revealed 20 proteins that are highly differentially expressed (DEPs) in the serum of gout patients relative to healthy controls. Notably, VEGF-A, MMP-1, TGF-α, and OSM were identified with high discriminative power by ROC. These high AUC values suggest that these proteins could be strong candidates for biomarkers in gout diagnostics. In addition, our analysis of synovial fluid revealed a distinct pattern in gout, with IP-10, VEGF-A, IL-8, and MIP-3β showing elevated levels compared to OA.

VEGF-A, a central mediator that regulates angiogenesis, plays a critical role in angiogenic, inflammatory, and bone-destructive processes in rheumatoid arthritis (RA) (18). Consequently, it may also contribute to inflammation in gout by increasing vascular permeability and recruiting inflammatory cells to the affected area. VEGFA can induce MMP-1 and other MMPs, which promote angiogenesis by degrading the extracellular matrix and enabling the migration of new cells within the synovium and the proliferation of new blood vessels (19). TGF-α belongs to the EGF family and promotes proliferation and differentiation in epidermal and epithelial cells (20). OSM is a versatile cytokine that participates in various inflammatory responses, including wound healing (21). Interferon Gamma-induced Protein 10 (IP-10) serves as a crucial biomarker in numerous diseases. It is a chemokine released in reaction to IFN-γ and plays a key role in the inflammatory response (22). IL-8 is a key mediator of the inflammatory response which acts as a chemoattractant and a potent angiogenic factor (23). MIP - 3β is a member of the chemokine family and plays a crucial role in the initiation, progression, and resolution of inflammatory responses.

The clinical implications of our findings are multifaceted. Firstly, the proteins identified as differentially expressed between gout and healthy controls, such as VEGF-A, MMP-1, TGF-α, and OSM, show high discriminative power and may serve as potential biomarkers for gout diagnosis. Elevated levels of these proteins in gout serum and synovial fluid suggest their involvement in the inflammatory process and tissue damage associated with the disease. The use of these biomarkers could aid in early detection and monitoring of disease progression, potentially leading to improved patient outcomes. Secondly, the similarity in proteomic signatures between gout and OA highlights the need for more specific biomarkers to differentiate between these conditions. The distinct patterns observed in gout, particularly with respect to IP-10, VEGF-A, IL-8, and MIP-3β, indicate their potential as differential diagnostic markers. These findings could facilitate more accurate diagnosis and personalized treatment strategies.

PPI on proteins with elevated expression levels in both gout serum and synovial fluid (SF) had revealed significant enrichment of pathways that are characteristic of gout. This approach underscores the complex interplay of these proteins in the pathogenesis of the disease and may provide insights into potential therapeutic targets. There is no doubt that the “Cytokine-cytokine receptor interaction” and “Chemokine signaling pathway” pathways were significantly enriched in gout. We also discovered that the “IL-17 signaling pathway” and “JAK-STAT signaling pathway” were enriched in gout. IL-17, a pro-inflammatory cytokine, plays a crucial role in mediating inflammation and immune responses, including arthritis. Studies have demonstrated that the IL-17A neutralizing antibody regulates monosodium urate crystal-induced gouty inflammation (24). Certain cytokines and molecules involved in the JAK-STAT signaling pathway may be overactivated or malfunctioning, leading to the excessive production of inflammatory mediators and the subsequent development and progression of gout symptoms (25). During gout flare-ups, the JAK2/STAT3 signaling pathway is activated, leading to increased expression of cytokines such as IL-6, IL-1β, and TNF-α in both the kidneys and joints (25). Interestingly, the “Cytosolic DNA-sensing pathway” and “AGE-RAGE signaling pathway” were also enriched in both serum and SF of gout. The “Cytosolic DNA-sensing pathway” plays a significant role in inflammation and arthritis. This pathway is involved in detecting the presence of cytosolic DNA, which can be released from damaged cells or pathogens, leading to the activation of immune responses (26). AGEs are formed through non-enzymatic reactions between sugars and proteins, and their accumulation in tissues can lead to chronic inflammation and tissue damage (27).

Based on the pathway enrichment analyses, several potential therapeutic targets emerge. For instance, the “Cytokine-cytokine receptor interaction” and “Chemokine signaling pathway” pathways are significantly enriched in gout. Targeting key components of these pathways, such as IL-17A and its receptors, has shown promise in preclinical models and clinical trials for rheumatoid arthritis and may also be beneficial for gout management (24, 28). The “IL-17 signaling pathway” and “JAK-STAT signaling pathway” are also enriched in both serum and synovial fluid in gout. Given the role of IL-17 and the JAK-STAT pathway in driving inflammation, inhibitors of these pathways represent potential therapeutic options. For example, targeting the JAK2/STAT3 signaling pathway could reduce the expression of pro-inflammatory cytokines like IL-6, IL-1β, and TNF-α, thereby alleviating gout symptoms and slowing disease progression. The “Cytosolic DNA-sensing pathway” is enriched in the serum of gout patients, suggesting that targeting components of this pathway, such as cGAS-STING signaling, could dampen excessive inflammation. Similarly, the “AGE-RAGE signaling pathway” is prominent in the synovial fluid of gout patients, indicating that inhibiting AGE formation or blocking the interaction between AGEs and RAGE could mitigate chronic inflammation and tissue damage.

One limitation of the present study is the relatively small cohort size, which could have led to an underestimation of the differences in protein levels between the gout and OA groups, given the stringent FDR correction applied. In reality, it is difficult to include a large number of patients with severe gout and OA with synovial fluid in basic science studies. Functional validation will be essential to identify the specific proteins and predicted pathways that play a role in the pathogenesis of gout. Another limitation of our study is that we only collected samples from patients with active gout, without including samples from different stages of gout, including stable gout. In future research, we plan to characterize the inflammatory profiles of gout at various phases, including stable gout, to provide a more comprehensive understanding of the disease.

Overall, this study provides a detailed exploration of the serum and SF proteomic signature in the context of gout, offering insights into the molecular mechanisms and potential therapeutic avenues for the condition. Further research is warranted to validate these findings and explore their clinical implications.

## Data Availability

The raw data supporting the conclusions of this article will be made available by the authors, without undue reservation.
