# Supplementary material for: A targeted proteomics screen reveals serum and synovial fluid proteomic signature in patients with gout
Source: Front Immunol. 2024 Nov 14;15:1468810. doi: 10.3389/fimmu.2024.1468810 (PMC11602490; doi:10.3389/fimmu.2024.1468810)
Supplement: Supplementary file 1 [file Table1.docx]

Supplementary Table 1. Baseline characteristics

|  | GS (n = 8) | OS (n = 8) | HS (n = 8) | GJ (n = 14) | OJ (n = 13) |
| --- | --- | --- | --- | --- | --- |
| Age, mean±SD | 49.6±11.1 | 51.3±6.54 | 42.9±8.8 | 52.4±10.7 | 56.5±9.3 |
| Male, n (%) | 6 (75) | 5 (63) | 5 (63) | 11 (79) | 6 (46) |
| CRP, mg/L | 48.7±4.6 | 4.6±3.8 | N/A | 30.4±23.7 | 19.2±24.0 |
| ESR, mm/h | 39.9±20.4 | 23.4±10.0 | N/A | 39.8±27.8 | 37.8±23.3 |
| Diabetes, n (%) | 1 (13) | 1 (13) | 0 | 1 (7) | 2 (15) |
| Hypertension, (%) | 1 (13) | 2 (25) | 1 (13) | 1 (7) | 3 (23) |

GS: the serum samples of gout patients; OS: the serum samples of OA patients; HS: the serum samples of healthy controls; GJ: the synovial fluid samples of gout patients; OJ: the synovial fluid samples of OA patients.
