# Supplementary material for: A targeted proteomics screen reveals serum and synovial fluid proteomic signature in patients with gout
Source: Front Immunol. 2024 Nov 14;15:1468810. doi: 10.3389/fimmu.2024.1468810 (PMC11602490; doi:10.3389/fimmu.2024.1468810)
Supplement: Supplementary file 2 [file Table2.docx]

Supplementary Table 2. The name of the protein corresponding to the gene in the PPI network.

| Protein | Gene |
| --- | --- |
| EGF | EGF |
| G-CSF | CSF3 |
| GM-CSF | CSF2 |
| HGF | HGF |
| IL-17F | IL-17F |
| IL-1β | IL1B |
| IL-2 | IL-2 |
| IL-27 | MYDGF |
| IL-6 | IL-6 |
| IL-7 | IL-7 |
| IL-8 | CXCL8 |
| IP-10 | CXCL10 |
| I-TAC | CXCL11 |
| LOX-1 | OLR1 |
| MCP-1 | CCL2 |
| MCP-2 | CCL8 |
| MCP-3 | CCL7 |
| MIP-1α | CCL3 |
| MIP-1β | CCL4 |
| MIP3B | CCL19 |
| MMP-1 | MMP-1 |
| MMP-12 | MMP-12 |
| OSM | OSM |
| TGF-α | TGFA |
| TWEAK | TNFSF12 |
| VEGF-A | COL18A1 |
